# Ultraviolet-ozone treatment reduces levels of disease-associated prion protein and prion infectivity

**DOI:** 10.1186/1756-0500-2-121

**Published:** 2009-07-06

**Authors:** Christopher J Johnson, PUPA Gilbert, Debbie McKenzie, Joel A Pedersen, Judd M Aiken

**Affiliations:** 1 Department of Comparative Biosciences, University of Wisconsin – Madison, 1656 Linden Dr, Madison, WI 53706, USA; 2 Department of Physics, University of Wisconsin – Madison, 1150 University Ave, Madison, WI 53706, USA; 3 Department of Soil Science & Molecular and Environmental Toxicology Center, University of Wisconsin – Madison, 1525 Observatory Dr, Madison, WI 53706, USA; 4 Current address: US Geological Survey, Biological Resources Division, National Wildlife Health Center, 6006 Schroeder Rd., Madison, WI. 53711, USA; 5 Current address: Alberta Centre for Prions and Protein Folding Diseases, Department of Biological Sciences, University of Alberta, Edmonton, Alberta, T6G 2M8, Canada; 6 Current address: Alberta Centre for Prions and Protein Folding Diseases, Department of Agricultural, Food and Nutritional Sciences, University of Alberta, Edmonton, Alberta, T6G 2M8, Canada

## Abstract

**Background:**

Transmissible spongiform encephalopathies (TSEs) are a group of fatal neurodegenerative diseases caused by novel infectious agents referred to as prions. Prions appear to be composed primarily, if not exclusively, of a misfolded isoform of the cellular prion protein. TSE infectivity is remarkably stable and can resist many aggressive decontamination procedures, increasing human, livestock and wildlife exposure to TSEs.

**Findings:**

We tested the hypothesis that UV-ozone treatment reduces levels of the pathogenic prion protein and inactivates the infectious agent. We found that UV-ozone treatment decreased the carbon and prion protein content in infected brain homogenate to levels undetectable by dry-ashing carbon analysis or immunoblotting, respectively. After 8 weeks of ashing, UV-ozone treatment reduced the infectious titer of treated material by a factor of at least 10^5^. A small amount of infectivity, however, persisted despite UV-ozone treatment. When bound to either montmorillonite clay or quartz surfaces, PrP^TSE ^was still susceptible to degradation by UV-ozone.

**Conclusion:**

Our findings strongly suggest that UV-ozone treatment can degrade pathogenic prion protein and inactivate prions, even when the agent is associated with surfaces. Using larger UV-ozone doses or combining UV-ozone treatment with other decontaminant methods may allow the sterilization of TSE-contaminated materials.

## Findings

Transmissible spongiform encephalopathies (TSEs, prion diseases) are a group of fatal neurodegenerative diseases that affect humans and a variety of domestic and wild mammals [1]. The disease agents responsible for TSEs are referred to as prions and are comprised primarily, if not solely, of a misfolded isoform of the prion protein, designated PrP^TSE^, derived from the normal cellular isoform of the protein (PrP^C^) [2]. Whereas PrP^C ^is susceptible to hydrolysis and degradation, the conformation adopted by PrP^TSE ^affords it protection from numerous aggressive treatments that inactivate conventional pathogens [3]. Incomplete sterilization of medical devices has resulted in iatrogenic transmission of human TSEs [4]. Development of effective prion decontamination methods represents an important goal in safeguarding human and animal health.

Ozone is a strong oxidant (*E*_*H*_^0 ^= 2.07 V) that chemically alters and inactivates numerous chemical contaminants and pathogens [5]. Ozone can be generated by corona discharge, cold plasma and ultraviolet (UV)-ozone devices [6]. In the case of UV-ozone generators, ultraviolet light at two wavelengths contributes to ozone generation and contaminant removal from surfaces: 185 nm photons dissociate O_2 _to O forming ozone (O_3_) via a radical reaction, and light at 254 nm excites bonds present in some organic contaminants [7]. UV-ozone treatment can be conducted at room temperature and pressure, is low-cost and has been successfully employed to remove carbon from Si microchip surfaces, x-ray optics and samples being prepared for elemental analyses (e.g., spectromicroscopy) [8-10]. Degradation of organic compounds by UV-ozone involves breakage of carbon-carbon bonds and CO_2 _evolution [7], and inactivation of proteins by ozone appears to occur, at least initially, via side-chain oxidation and structural rearrangement [11]. Although UV-based systems produce much less ozone and require substantially longer exposure times than other generators, spectromicroscopic analyses have demonstrated that UV-ozone effectively removes carbon from samples while preserving the ultrastructure of treated samples [9,10]. In the present study, we investigated the degree to which UV-ozone inactivated prions deposited on Si wafers or associated with quartz or montmorillonite clay (Mte) surfaces, using conditions identical to those that remove carbon from spectromicroscopy samples.

The Hyper strain of hamster-passaged transmissible mink encephalopathy agent (HY) was used in all experiments [12]. Brain homogenate (BH), 10% w/v in ddH_2_O, was either deposited on inert Si wafer substrates (8 cm × 1 cm × 500 μm) or, for studies examining degradation of PrP^TSE ^bound to particle surfaces, was allowed to adsorb to particles using published protocols [13]. Briefly, following clarification by centrifugation, 30 μL HY BH was incubated for 2 h in 10 mM NaCl with 0.5 or 3.2 mg of Mte or quartz microparticles, respectively, or in the absence of particles for control samples. All solutions were air-dried overnight and UV-ozone treatment was initiated the following day. Samples were prepared such that UV-ozone exposure was terminated on the same day for all samples. Aliquots of all particle-free samples (0–8 weeks treatment) were prepared for total carbon analysis (dry ashing method, Leco CNS-2000 analyzer) [14], immunoblotting using monoclonal antibody 3F4 and published protocols [13], and intracerebral inoculation into Syrian hamsters (*Mesocricetus auratus*, cared for in accordance with institutional animal care protocols). Samples containing particles were prepared for immunoblotting. Digestion of HY BH with 50 μg·mL^-1 ^proteinase K (PK) for 30 minutes indicated the initial presence of PrP^TSE ^in the starting material.

UV-ozone exposure was conducted in an ashing oven consisting of enclosed metal housing equipped with a custom-made, cold-cathode, low-pressure, grid mercury lamp (producing UV radiation with wavelengths of 185 and 254 nm; 1.5 mW·cm^2 ^at 1 in) mounted on a 5 cm × 15 cm Alzak reflector (Jelight Co., Irvine, CA). Dry air (<1% relative humidity, 20°C) was pumped into the oven (1.2 L·min^-1^) to purge CO_2 _and replenish O_2 _for ozone generation. Silicon substrates with dried brain homogenate were placed 2 mm from the lamp and ashed for 0–8 weeks. After ashing, residual brain material was removed from substrates by agitation in phosphate buffered saline (PBS, for carbon analysis, immunoblotting and bioassay) or 10× SDS-PAGE sample buffer (100 mM Tris pH 8.0, 10% SDS, 7.5 mM EDTA, 100 mM dithiothreitol, 30% glycerol, for immunoblotting only) at 95°C. Material was removed from the silicon surface until the substrate appeared visibly clean and mirror-like. Sample buffer extraction of residual PrP^TSE ^is a harsh treatment that denatures the protein and ranks among the most effective known means of removing PrP^TSE ^from surfaces [13,15]. Test extractions with 10× SDS-PAGE sample buffer at 95°C or PBS at 20°C were equally effective on removing both ashed and unashed material from Si wafers (data not shown). For the non-ashed control sample (0 weeks), brain material was removed from the Si substrate following overnight drying.

**Figure 1:**
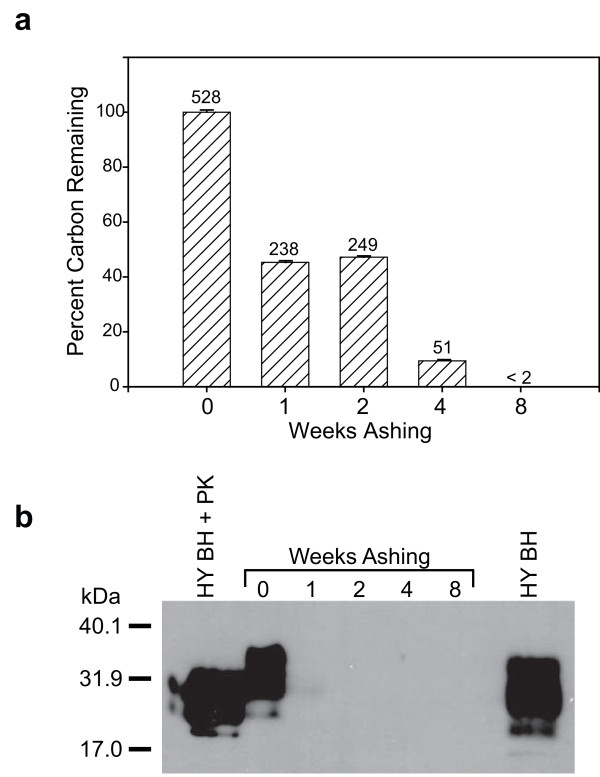
**UV-ozone treatment decreases carbon and PrP^TSE ^levels**. (**a**) Total (organic and inorganic) carbon was measured following 0, 1, 2, 4 or 8 weeks of UV-ozone treatment. Bars represent means ± one standard deviation; numerical values above bars indicate the mean mass of carbon remaining in μg. Experiment is representative of two independent replicates. (**b**) Immunoblot analysis of prion protein following ozone ashing for the indicated time period. Hyper-infected brain homogenate (HY BH) and HY BH treated with 50 μg·mL^-1 ^proteinase K (PK) demonstrate the presence of PrP^TSE ^before ashing. Immunoblot used anti-prion protein antibody 3F4.

UV-ozone ashing of brain homogenate (BH) from TSE-infected hamsters resulted in a time-dependent loss of carbon (Figure 1a). Approximately 50% of the carbon was lost after 1 week of ashing (initial value of 528 ± 2 μg was reduced to 238 ± 1 μg). Interestingly, no decrease in carbon content occurred during the second week of UV-ozone treatment (249 ± 1 μg). At 4 weeks, the carbon content had decreased to ~10% of the initial concentration (51 ± 2 μg). Carbon was not detected after 8 weeks of ashing, indicating that < 2 μg C remained.

Non-linear carbon loss in HY BH samples could indicate resistance of a subset of biomolecules to UV-ozone degradation. Previous work has shown that DNA and RNA are substantially more susceptible to ozone attack than proteins [16] and dried proteins are particularly resistant to ozone action [11]. Experiments investigating sample thickness, biomolecule composition and carbon loss kinetics may provide insight into the cause of the observed non-linearity in carbon loss from BH.

UV-ozone treatment reduced PrP^TSE ^levels in a time-dependent manner (Figure 1b). After one week of ashing, prion protein immunoreactivity was reduced to nearly undetectable levels by immunoblotting and after two weeks, levels were below the limits of immunoblotting detection. Our previous work has shown that similar reductions in immunoreactivity correspond to at least a 200-fold loss of PrP^TSE ^[17]. As expected, prion protein levels remained below the limit of detection in samples exposed to either 4 or 8 weeks of UV-ozone.

Intracerebral inoculation of samples into hamsters allowed direct assessment of the degree to which UV-ozone treatment diminished the infectious titer of ashed sample extracts. Table 1 presents the results obtained from 43 hamsters inoculated with UV-ozone treated (11 animals) and untreated infectious BH at various dilutions (32 animals), and the time to onset of clinical symptoms after inoculation. Weanling hamsters were intracerebrally dosed with ozone-treated material or a dilution series of the starting BH as a control on which to base estimates of remaining infectious titer in ashed samples. Each 50 μL sample of undiluted, unashed BH contained 10^6^–10^7 ^infectious units [12]. Based on the dilution series, ~10^2 ^infectious units of TSE agent remained in BH after 4 weeks ashing (Table 1). With material which had been subjected to 8 weeks of ashing, two of seven inoculated hamsters did not succumb to disease within a 365-day period (Table 1). UV-ozone clearly reduced the titer of ashed TSE agent. Precise determination of low prion titers is challenging [18], but the bioassay data indicate that the UV-ozone ashing conditions used here reduced TSE agent titer by at least a factor of 10^5 ^and possibly more.

**Table 1:** UV-ozonation decreases infectious TSE titer and increases disease incubation.

**Inoculum**	**Positive/Total Animals**	**Onset of Clinical Symptoms (dpi)**	**Estimated Titer (ID_50 _per 50 μL dosage)**	**Approximate Reduction in Titer**
**Dilutions of Starting Material**				

HY BH (10% w/v)	4/4	67 ± 0*	10^6^-10^7^	Not applicable

HY BH (10^2 ^dilution factor)	8/8	86 ± 0*	10^4^-10^5^	10^2^

HY BH (10^4 ^dilution factor)	8/8	107 ± 0*	10^2^-10^3^	10^4^

HY BH (10^6 ^dilution factor)	2/8	141, 156^†,‡^	0–10^1^	10^6^

PBS	0/4	>365^‡^	0	Not applicable

				

**UV-ozone Treated Material**				

HY BH (10% w/v) ashed 4 weeks	4/4	120 ± 10*	10^1^-10^2^	10^5^

HY BH (10% w/v) ashed 8 weeks	5/7	127, 136, 136, 136, 141^†,‡^	1–10^1^	10^6^

* Mean days post inoculation (dpi) ± SD to onset of clinical symptoms of TSE infection

^† ^Number of dpi to onset of clinical TSE symptoms for each clinically-affected animal in the group

^‡ ^Animals showing no clinical signs of TSE infection were sacrificed 365 days post-inoculation

**Figure 2:**
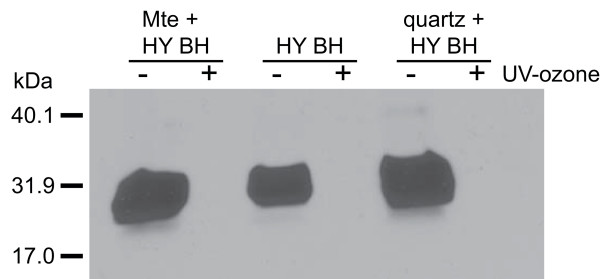
**Susceptibility of Mte or quartz bound PrP^TSE ^to UV-ozone degradation**. Immunoblot analysis of PrP immunoreactivity following 7 days of UV-ozonation (+) or incubation without UV-ozone (-) of Hyper-infected brain homogenate (HY BH) or HY BH bound to montmorillonite clay (Mte) or quartz. Immunoblot used anti-prion protein antibody 3F4.

To test whether UV-ozone is capable of degrading prions bound to surfaces, we bound HY BH to two particles with different surfaces properties, namely Mte and quartz [13], and subjected both particle- and nonparticle-associated HY BH to 1 week of UV-ozone treatment (Figure 2). Samples prepared and incubated identically, but not exposed to UV-ozone, served as controls. Following ozonation or incubation, all samples were extracted with 10× sample buffer, a harsh treatment capable of removing approximately 95% of PrP^TSE ^from mineral surfaces [13,15]. No prion protein immunoreactivity was detectable in any of the UV-ozone treated samples suggesting UV-ozone is capable of degrading PrP^TSE ^bound to surfaces.

Our results indicate that, in a controlled setting, that UV-ozone treatment degrades PrP^TSE ^and inactivates prions. The relative contributions of ozone and ultraviolet light toward reducing PrP^TSE ^levels are difficult to ascertain in our system as UV light is required for ozone production. We hypothesize that much of the observed degradation and inactivation resulted from ozone exposure for the following reasons. First, a reduction in carbon content, such as that observed in Figure 1a, is a characteristic effect of ozone and not of UV radiation [7]. Second, in aqueous media, proteins in general and TSE infectivity in particular, resist large doses of UV radiation [19], and dried biomolecules, such as those in our system, exhibit more resistance to UV inactivation than do wet samples [20,21].

We consider it unlikely that UV-ozone treatment caused irreversible binding of PrP^TSE ^to all tested surfaces (viz. Si, Mte and quartz), due to (1) substantial differences in surfaces properties among the materials, (2) previous reports indicating that proteins are more easily removed from surfaces following ozonation [22,23] and (3) the efficacy of 10× sample buffer in removing avidly-bound PrP^TSE ^from mineral surfaces [13,15]. Bioassay of the ozone-treated Mte or quartz bound PrP^TSE ^will clarify the extent to which UV-ozone affects surface-bound prions.

In the present study, we employed a gentle UV-ozonation approach. Use of more aggressive UV-ozone treatment (e.g., by use of a higher wattage lamp), other techniques that produce higher ozone concentrations, ozone in combination with either other species of reactive oxygen or with other decontaminants might more effectively diminish prion titers. The hamster TSE strains, HY and 263K, are structurally related [24]; HY is as resistant to guanidine denaturation and PK digestion as 263K and Sc237 strains [25,26]. The stability of the HY strain and its degradation by UV-ozone suggest that this method may have utility in decontaminating other prion strains, and investigation into UV-ozonation to decontaminate human strains or prions bound to stainless steel surfaces, as a model for surgical instruments, is warranted.

## Abbreviations

BH: brain homogenate; dpi: days post-inoculation; HY: Hyper strain of hamster-passaged transmissible mink encephalopathy agent; Mte: montmorillonite clay; PAGE: polyacrylamide gel electrophoresis; PBS: phosphate buffered saline; PK: proteinase K; PrP^C^: cellular prion protein; PrP: prion protein; PrP^TSE^: disease-associated prion protein; TSE: transmissible spongiform encephalopathy; UV: ultraviolet.

## Competing interests

The authors declare that they have no competing interests.

## Authors' contributions

CJJ and PUPAG conceived the study, designed and performed the experiments, analyzed the data and wrote the manuscript. DM, JAP and JMA analyzed the data and wrote the manuscript. All authors read and approved the final manuscript.
